# Treatment timing shifts the benefits of short and long antibiotic treatment over infection

**DOI:** 10.1093/emph/eoaa033

**Published:** 2020-11-23

**Authors:** Erida Gjini, Francisco F S Paupério, Vitaly V Ganusov

**Affiliations:** 1 Mathematical Modeling of Biological Processes Laboratory, Instituto Gulbenkian de Ciência, Rua da Quinta Grande, 6, Oeiras, 2780-156, Portugal; 2 Departamento de Informática, Faculdade de Ciências, Universidade de Lisboa, Campo Grande, Lisbon, 1749-016, Portugal; 3 Department of Microbiology, University of Tennessee, Knoxville, TN 37996, USA

**Keywords:** antibiotic resistance, infection dynamics, treatment duration and timing, immunity-resistance tradeoff

## Abstract

Antibiotics are the major tool for treating bacterial infections. Rising antibiotic resistance, however, calls for a better use of antibiotics. While classical recommendations favor long and aggressive treatments, more recent clinical trials advocate for moderate regimens. In this debate, two axes of ‘aggression’ have typically been conflated: treatment intensity (dose) and treatment duration. The third dimension of treatment timing along each individual’s infection course has rarely been addressed. By using a generic mathematical model of bacterial infection controlled by immune response, we examine how the relative effectiveness of antibiotic treatment varies with its timing, duration and antibiotic kill rate. We show that short or long treatments may both be beneficial depending on treatment onset, the target criterion for success and on antibiotic efficacy. This results from the dynamic trade-off between immune response build-up and resistance risk in acute, self-limiting infections, and uncertainty relating symptoms to infection variables. We show that in our model early optimal treatments tend to be ‘short and strong’, while late optimal treatments tend to be ‘mild and long’. This suggests a shift in the aggression axis depending on the timing of treatment. We find that any specific optimal treatment schedule may perform more poorly if evaluated by other criteria, or under different host-specific conditions. Our results suggest that major advances in antibiotic stewardship must come from a deeper empirical understanding of bacterial infection processes in individual hosts. To guide rational therapy, mathematical models need to be constrained by data, including a better quantification of personal disease trajectory in humans.

**Lay summary**: Bacterial infections are becoming more difficult to treat worldwide because bacteria are becoming resistant to the antibiotics used. Addressing this problem requires a better understanding of how treatment along with other host factors impact antibiotic resistance. Until recently, most theoretical research has focused on the importance of antibiotic dosing on antibiotic resistance, however, duration and timing of treatment remain less explored. Here, we use a mathematical model of a generic bacterial infection to study three aspects of treatment: treatment dose/efficacy (defined by the antibiotic kill rate), duration, and timing, and their impact on several infection endpoints. We show that short and long treatment success strongly depends on when treatment begins (defined by the symptom threshold), the target criterion to optimize, and on antibiotic efficacy. We find that if administered early in an infection, “strong and short” therapy performs better, while if treatment begins at higher bacterial densities, a “mild and long” course of antibiotics is favored. In the model host immune defenses are key in preventing relapses, controlling antibiotic resistant bacteria and increasing the effectiveness of moderate intervention. In order to improve rational treatments of human infections, we call for a better quantification of individual disease trajectories in bacteria-immunity space.

## INTRODUCTION

Treatment of bacterial infections has for many decades relied on the use of antibiotics. Although antibiotics have saved many lives and enabled uncountable medical practices, their widespread use in human and animal populations has led to the rise of antibiotic resistance, posing now a threat to human health and modern medicine [1]. Of particular concern is the rise of multidrug-resistant bacteria, favored by the use of wide-spectrum antibiotics especially in clinical settings [2, 3]. To confront these challenges, much research has been devoted to understand the molecular, genetic and non-genetic mechanisms leading to drug resistance in bacteria [4–6], their population dynamic and interplay with treatment strategies [7–9]. While alternative approaches such as anti-virulence therapies [10], or therapies that stimulate the host’s capacity to deal with infection [11], are also being considered, with their own potential limitations [12], reducing antibiotic use remains essential in addressing the antibiotic resistance crisis. In this context, it is important to understand the rational principles by which antibiotics succeed and fail in clearing infections, and whether and when aggressive or moderate treatments are superior. It is here that mathematical models, alongside clinical trials and surveys, can help.

The conventional wisdom of treating infections with high-antibiotic doses (aggressive treatment) [13] to avoid resistance emergence has recently been challenged [14, 15], on the basis of evolutionary arguments showing a bigger risk of resistance selection with more aggressive treatments (see [7] for a review). Many studies including clinical trials have by now shown that for some infections shorter treatment is not inferior to the longer ones and that longer treatment may in fact result in failure if resistant bacteria are already present when treatment starts [16–20]. This issue is now recognized in clinical practice and checklists of improving antibiotic prescribing have been suggested [21].

On one hand, clinical studies have been concerned mainly with optimal duration of therapy, on the other, the multiple mathematical studies addressing the question of optimal antibiotic treatment of bacterial infections [22–25], have focused mainly on the dosing dimension, with a few studies exploring duration [26] and timing of treatment [27]. While these studies have highlighted the various complexities in optimal treatment, typically two axes of aggression have been conflated: treatment length and treatment intensity, and a single criterion for defining optimality, e.g., resistance emergence or selection, has been often considered.

**Fig. 1:**
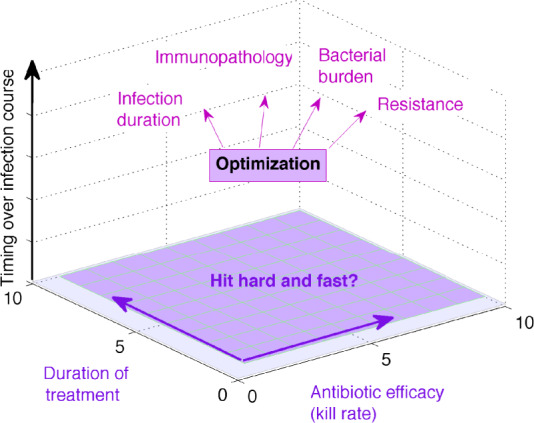
Antibiotic treatment and optimization in a multi-dimensional context. In this study, we evaluate treatment of an infection in a 3D space spanned by antibiotic strength, duration and timing, along several target criteria of clinical and epidemiological importance. Optimizing the parameters of antibiotic treatment is likely to involve several alternative goals (e.g. reducing bacterial load or minimizing antibiotic resistance), but from the perspective of the patient and treating physician, successful treatment generally means rapid reduction in symptoms and no disease relapse after treatment. Some of these will be more possible to reach than others, depending on the stage of infection and characteristics of the patient starting treatment.

Here, we develop a more comprehensive approach to address the treatment–infection interplay along several additional dimensions (Fig. 1). We use a simple mathematical model of a bacterial infection that is controlled by the immune response, capturing not only dynamic build-up of host defenses as a result of infection but also baseline immune competence in terms of efficiency for controlling low pathogen numbers. This model element adds a dose-dependency to the natural outcome of infection, which previous treatment models have not accounted for or emphasized. Furthermore, this feature enables the contextualization of other processes such as temporary immune suppression or vaccination, which may interfere with the need for and effect of antibiotic treatment.

With this model, we investigate the role of treatment timing, intensity and duration, across different metrics of successful treatment. We show that duration of antibiotic treatment, antibiotic efficacy (defined as the antibiotic kill rate) and treatment timing interact non-linearly to determine the final outcome, and that optimal regimes vary with target criterion for optimization even at a single host level. While our results suggest that it is unlikely that one optimal treatment duration exists, they also indicate many parameter regimes where short and long treatments are both successful, and point to useful gradients for future empirical and clinical investigation.

## THE MODEL

We describe a typical bacterial infection, where the pathogen grows, stimulates host immunity and ultimately is controlled by a sufficiently expanded immune response. Infection is initiated by the drug-sensitive bacteria (*B_s_*) which grow exponentially at a rate *r*, and experience density-dependent limitation via a carrying capacity *C*, similar to previous models [26]. Drug-resistant bacteria (*B_r_*) may not be present initially but can be generated via mutation with rate *m*. We assume that resistance bears a cost *γ* which reduces the growth rate of the drug-resistant sub-population to r(1−γ) and prevents it to overcome *B_s_* in the absence of treatment. For bacterial infections of humans, it is generally poorly understood which types of immunity—innate or adaptive—are most important in the control of the specific infection, and in general, they do interact via complex bidirectional feedbacks. Here, we do not make an explicit distinction between innate and adaptive immunity, but rather implement two *modes* of immune control: (i) one *static* immune response, *I*, which is more effective at low pathogen numbers and is assumed constant and (ii) a dynamic immune response, *E*, that displays infection-coupled kinetics and is triggered at higher pathogen loads. Both of these defenses (capturing roughly constitutive and inducible mechanisms) exert a negative feedback on infection, albeit each one at a different characteristic dynamic range. The major assumption for the static immune response is that the per-capita pathogen killing rate it displays goes down with pathogen level *B*. This saturated strength of killing could mechanistically result from constraints of handling time, and minimal ratios of respective cell numbers required for effective killing, for example, a critical number of phagocytes (e.g. neutrophils or macrophages) per bacteria [28]. The maximal kill rate per unit bacteria per unit static response, in the limit of very low pathogen numbers, is captured by *δ*.

For the dynamic immune response, we assume saturating stimulation by pathogen load; its growth gets triggered after total bacteria reach some density, defined by a half-saturation constant *k*. The bacterial density at which the specific immune response grows at one-half of its maximum rate has an intermediate value between the initial bacterial density and carrying capacity, similar to [22]. When bacterial density is high, the immune response increases at maximal rate *σ* until the infection is cleared. Killing of bacteria by this immune response is assumed to occur at a rate directly proportional to the magnitude of the immune response, with a killing rate constant *d*, equal for sensitive and resistant bacteria, similar to [22, 29]. The initial immunity level is given by $E(0)=E_{0}$, higher levels of which, are expected to reduce from the start of the net growth rate of bacteria within the host.

Some of these dynamic motifs in the immune response against bacterial infections appear also in other modeling studies. For example, the saturating stimulation of the antigen-dependent immune response (*E* in our model) has been used also by [22] when implementing their pathogen density-dependent response. The feature of our other immune response (*I*), whereby the rate at which bacteria are killed saturates as the bacterial load increases, has been used also in the study by [29], in their immune response models 2 and 4, but [29], in addition, include also a pathogen-independent growth dynamics of such response that is triggered upon onset of infection, while here we take *I* roughly constant over infection.

With these biological features, our model is given by the following ordinary differential equations:

(1) $$\frac{dB_{s}}{dt}=rB_{s}(1-\frac{B}{C})-dEB_{s}-\delta I\frac{B_{s}}{1+hB}-A_{m}B_{s}\eta (t),$$

 

(2) $$\frac{dB_{r}}{dt}=r(1-\gamma )B_{r}(1-\frac{B}{C})-dEB_{r}-\delta I\frac{B_{r}}{1+hB},$$

 

(3) $$\frac{dE}{dt}=\sigma E\frac{B}{B+k},$$

where $B=B_{s}+B_{r}$ and η(t) are step-function, varying between 0 and 1 to reflect antibiotic treatment during a given time interval ($\tau_{1}\leq t\leq \tau_{1}+\tau_{2}$). Notice that the deterministic equation for *B_r_* is only active after this sub-population has been generated within host, assuming infections start with the drug-sensitive bacteria. Following the hybrid approach and deterministic approximation proposed by [30], that treats population growth as deterministic, but the time of first appearance of mutants as stochastic, similar to the original Luria–Delbruck model [31], for resistance emergence, we track the probability of no-emergence by time *t*: $P(t)=e^{-m\int_{0}^{t}B_{s}(x)dx}$. Thus, we simulate the arrival of the resistant sub-population when this probability hits a deterministic threshold, in our case *P *=* *0.5, which implies our simulated emergence time, *t*_em_, corresponds to the median arrival time. Then *B_r_* is initialized at level *B*_em_, and subsequently let to follow the deterministic growth of Eq. (2) during *t* > *t*_em_ (see Supplementary Figs S1 and S2 for an illustration). Finally, our model implements an extinction threshold *B*_ext_ when either bacterial compartment falls below this level, in line with previous studies [23, 27]. This prevents too low bacterial densities from artificially bouncing back after a decline. Numerical predictions of the model are carried out using MATLAB.

## TREATMENT AND METRICS OF SUCCESS

Typically, the timing of treatment relative to the natural infection course is not known. Previous models have assumed that treatment occurs throughout infection [23] or when infection reaches a peak [22], which imposed strong constraints on the model dynamics. In [27], treatment timing is explored in more detail, showing that it has dramatic consequences on the effectiveness of antibiotic treatment, both when it is given as a fixed regimen, and especially when it is administered in a more adaptive fashion, coupled to infection dynamics. Similar to [27], here, we also couple treatment timing to a threshold during infection, and vary it to accommodate different levels of host tolerance to the infection burden. It is possible, though, that other factors such as the degree of inflammation trigger the onset of symptoms, and thus, the seeking of treatment by the patient. Antibiotic treatment starts when the total bacterial density exceeds value Ω, lasts for *τ*_2_ days, and is described by the step-function *η*. Antibiotics increase the death rate of drug-sensitive bacteria *B_s_* by the rate *A_m_* (antibiotic kill rate), while the *B_r_* strain is assumed fully drug-resistant, making our study a conservative investigation of a ‘worst-case’ scenario of empiric therapy.

To investigate whether alternative treatment goals [15] conflict with optimal treatment (Fig. 1), we track several instantaneous or cumulative measures such as the density of bacteria *B*(*t*) or the level of immunity *E*(*t*) after treatment, the duration of infection, cumulative bacterial load (area under the curve, ${\text{AUC}}_{B}$) and total resistance burden (${\text{AUC}}_{R}$). By virtue of our hybrid modeling approach, our resistance burden is an average combined measure that takes into account the probability of emergence in an infection, and the subsequent mutant growth and selection. In addition, we also consider a simple and clinically observable criterion, namely the resolution of symptoms, which may be more readily linked with comparative surveys.

## MODEL PARAMETERS

As our model shares some features with previous within-host models [23, 25, 32], we adopt a similar range of parameters. Rather than mimicking a specific host–bacterial species scenario, our formulation is based on classical dynamic motifs of host–pathogen interaction, and is likely to apply to extra- and intra-cellular bacterial infections. Quantitative details of bacterial infections of humans are nearly absent, and therefore, we chose model parameters to constrain the overall bacterial dynamics in the absence of treatment. Replication rates of bacteria *in vivo* are assumed in the range 3–10 per day, consistent with previous modeling studies and empirical estimates for bacteria like *Staphylococcus aureus*, *Escherichia coli* and *Pseudomonas aeuriginosa* [33]. Bacterial net growth rate is reduced within a few days since infection [34], which in our model is obtained via rapidly activating and expanding antigen-dependent immune response [35–37].

Antibiotic kill rates have been accurately measured for several drugs *in vitro* [38] but not in humans, and thus were varied within expected range, to comprise both sub-inhibitory and supra-inhibitory effects on the pathogen population during infection. Modeling only kill rates instead of explicit drug concentrations and concentration-dependent killing via Hill functions, as done by previous studies [22, 25, 29], has, for us, a three-fold advantage: it reduces the number of heuristic parameters to specify in an *in vivo* infection model, enables different antibiotics at different concentrations to achieve the same effect, and helps to strengthen our focus on the three main axes of treatment. In particular, we rely on the assumption that antibiotic concentrations to achieve effective killing of sensitive bacteria are above the MIC measured *in vitro*, while for the resistant strain, the MIC is assumed to exceed the concentration that could be feasibly administered to the patient, thus leading to a zero effective kill rate of *B_r_* in the model. Specific values of parameters are given in Table 1.

**Table 1:** Parameters of the mathematical model.

Symbols	Parameter	Default	Typical range	References
				
* r*	Growth rate of bacteria	3	3–10/day	[27, 33]
* C*	Carrying capacity of bacteria	10^9^ cell/ml	*C* > *k*	[23]
* γ*	Fitness cost of resistant bacteria	0.2	0.05–0.2	[22]
* B*_0_	Initial inoculum	10^4^ cell/ml	$B_{0}<k$	[25, 32]
* σ*	Maximal growth rate of the immune response	1/day	1–4	[27]
* k*	Half-saturation constant for antigen-dependent immunity	10^8^ cell/ml	*k* < *C*	[27]
* d*	Elimination rate of bacteria by host immunity	$1\times 10^{-6}/\text{day}$	$10^{-6}-10^{-4}$	[39, 22]
* E*_0_	Initial immunity	$0.5\times E_{\mathrm{crit}}$	$E_{0}<E_{\mathrm{crit}}\equiv \frac{r}{d}$	[32]
	Static-immunity parameters			
* δ*	Effectiveness of static immune response	1	1–50	Illustrative of variable baseline immunity
* I*	Background immune response that acts at low pathogen levels	1	Scaled	Assumed constant during infection
* h*	Handling time per bacterial cell/ml by one unit of the static immune response	0.005	Fixed	To reproduce threshold *B*_0_ below 10^4^ leading to clearance [40]
* m*	Mutation rate conferring drug resistance per sensitive cell	$10^{-5}$	$10^{-6}-10^{-4}$	[41]
* B*_ext_	Pathogen extinction threshold (=*B*_em_, emergence threshold)	10	1−10	[23]
* P*_em_	Probability threshold for resistant mutant to emerge	0.5	[0,1]	[30]
* *Ω	Bacterial density threshold leading to symptoms and onset of treatment	10^6^ cell/ml	$10^{5}-10^{7}$	Sets treatment onset τ_1_, coupled to infection course before the peak, i.e. when B(t)=Ω [42]
* A_m_*	Antibiotic kill rate of drug-sensitive bacteria (efficacy)	1/day	0.1−10/day	[27]
* τ*_2_	Duration of antibiotic treatment	3, 7 days	1−14 days	[43]

These parameter values were chosen to generate an infection that would be self-limiting over a 10–20 day period, and display similar numerical range to other models of acute infections [22, 23, 27].

## RESULTS

### Infection outcomes and dependency on initial conditions

**Fig. 2:**
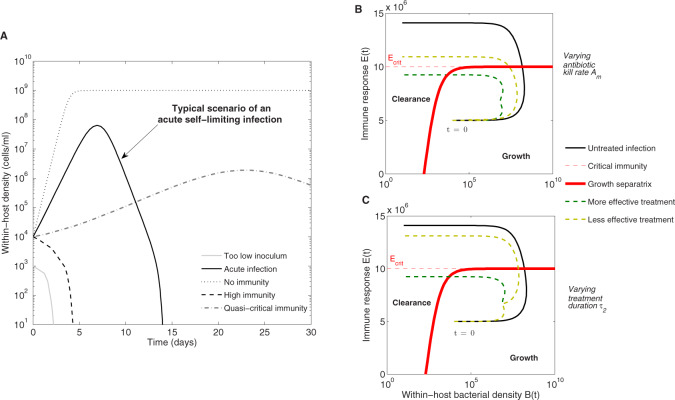
Infection model scenarios and individual trajectories in bacteria-immunity space. (**A**) Biological outcomes in the absence of treatment. For too low inoculum sizes *B*_0_, infection does not occur, as bacteria are successfully controlled by the static baseline immunity. For higher inoculum sizes, infection may still be prevented if initial effector response *E*_0_ is high enough. For sub-critical *E*_0_ and generally *k *<* C*, an acute infection is likely to occur. When *E *=* *0, or if k≫C, then infection persistence is possible. The dynamics of susceptible and resistant bacteria in such a situation follow logistic growth up to carrying capacity *C*, where both *B_s_* and a minority of *B_r_* may coexist. Parameters as in Table 1, unless otherwise stated. (**B** and **C**) Acute infection as a 2D trajectory, pairing *B*(*t*) and *E*(*t*) at each time point, in the absence and presence of treatment. The red line denotes (*B*, *E*) combinations that inhibit bacterial growth (ϕ=0), thus all initial conditions of the system to the left of the red curve lead to bacterial clearance, and all initial conditions to the right lead to an acute infection (typical course in black). (B) Effect of treatment, when changing the antibiotic kill rate *A_m_* (given in per day units). (C) Effect of treatment, when changing the treatment duration *τ*_2_. Bacterial density triggering the onset of symptoms is assumed to be $\Omega =10^{6}$. Treatment benefits can be obtained with either appropriate dosing or duration, but may come at the cost of resistance selection. In (B), two antibiotic kill rates, *A_m_* = 4 versus *A_m_* = 2 (with $\tau_{2}=10$), are depicted in green and yellow dashed lines, respectively, and in (C), a long treatment of $\tau_{2}=10$ days, and short one of $\tau_{2}=5$ days (with *A_m_* = 4) are plotted in green and yellow. The rest of parameter values are as in Table 1, except for: r=10,,δ=20, with initial conditions $B_{0}=10^{4}$ and $E_{0}=0.5E_{\mathrm{crit}}$.

Despite its simplicity, the model generates a variety of behaviors (Fig. 2A). In our analysis, we focus on acute infections generated in the presence of an immune response and on the role of immunity in the dynamics of antibiotic resistance. We first investigate the effect of pathogen inoculum size and initial immune response parameters on infection outcomes without treatment. It is straightforward to see from the model that, for $B_{0}\ll C$, the initial per-capita growth rate of bacteria within-host in the absence of intervention is given by:

(4) $$\phi =\frac{1}{B}\frac{dB}{dt}{|}_{t=0}=r-dE_{0}-\frac{\delta I}{1+hB_{0}}=d\left(E_{\mathrm{crit}}-E_{0}\right)-\frac{\delta I}{1+hB_{0}},$$

where *E*_0_ reflects the initial level of specific immunity, *B*_0_ the inoculum size and $E_{\mathrm{crit}}=r/d$. Under negligible background static immunity (*I *=* *0) the last term in Eq. (4) vanishes, and the initial growth or decline of bacteria will depend only on initial levels of the inducible response $E_{0}.$ More precisely, there is a critical value *E*_crit_, such that if $E_{0}>E_{\mathrm{crit}}$, bacteria immediately decline and there is no infection. If $E_{0}<E_{\mathrm{crit}}$, bacterial growth is possible leading to an acute infection. However, background static immunity (*I *>* *0) reduces the net bacterial growth rate, and this reduction is larger for smaller bacterial numbers *B*_0_. Only if *B*_0_ is high enough, will there be growth of the initial inoculum. This is a pattern expected across many infections, including those caused by *Streptococcus pneumoniae*, *Listeria monocytogenes*, *E.coli* and *S.aureus*. Notably, depending on the magnitude and potential fluctuations of other parameters such as *δ*, *I* and *h*, this dose-dependent effect can be different not only across hosts but also within the same host at different time points. For example, as a result of prior infection by the influenza virus, *I* or *δ* can transiently go down, which would enhance the susceptibility of this host to infection by lower doses of bacterial pathogens, for example, *S. pneumoniae*, a phenomenon that has been reported [40]. Similarly, the effects of vaccination in enhancing phagocytosis efficiency could also be implemented in this background static immunity as a higher *δ*, which would make the particular host refractory to higher doses of pathogen challenge. Thus, an important feature of the model is the mechanistic dose-dependent risk of infection, above the stochastic extinction threshold, and this is expressed by a critical line connecting *E* and *B* values, including (*E*_0_, *B*_0_) combinations, where ϕ=0. This separatrix defines growth and clearance regimes, mediated by initial immunity and initial pathogen inoculum, and may shift for different within-host parameters (Supplementary Fig. S3). We remark to emphasize that the stochastic extinction threshold and the dose-dependent risk of infection refer to different phenomena. While with respect to the stochastic extinction threshold, the net growth rate of bacteria is always the same for any inoculum size *B*_0_, with respect to the growth separatrix defined by static immunity, the net growth or decline rate of bacteria is different for different inoculum sizes.

Thus, acute infections start with sufficiently high inocula of drug-sensitive bacteria and relatively low levels of immunity. As the bacteria grow, the resistant mutant sub-population is generated and starts to increase, albeit at a lower rate, due to its fitness cost. Both the resistant and susceptible populations stimulate the dynamic immune response, which effectively grows after *B*(*t*) > *k*. In the absence of drug treatment, infections are self-limiting, brought under control by super-critical host defenses (e.g. E>r/d), and the resistant population occupies only a small part of the total load (Supplementary Fig. S1A).

Acute infection dynamics can also be represented as a 2D trajectory in bacteria-immunity space, involving co-variation of *B*(*t*) and *E*(*t*) (Fig. 2B and C), which helps to contextualize the crucial role of an intervention. During a typical infection course, sufficiently high inoculum sizes (on the right of the red separatrix), before being driven back to low numbers, make a big excursion in phase space and stimulate dynamic immune response. Sometimes antibiotic treatment is necessary to reduce this excursion, or to drive bacteria to the clearance zone with lower than absolutely critical immune activation; this limits pathology and can be life-saving for the host. When antimicrobial treatment is started, the sensitive population declines due to the drug-mediated killing, which allows the resistant population to increase. Depending on immune response levels, population composition and treatment parameters, there can be growth, relapse or clearance, as a result of treatment. The beneficial effects of treatment can be obtained with appropriate antibiotic kill rates (Fig. 2B) or appropriate duration (Fig. 2C); typically a good match between the two is key, but not easy to find (Supplementary Fig. S4). Although one treatment may seem more effective in disease-space, because a smaller *B* – *E* area is covered, the time to ‘travel’ in disease space may be faster in alternative treatments, leading to faster recovery and also less resistance selection (see Supplementary Fig. S4). How the beneficial effects of treatment can be amplified or complicated by multiple factors, is what we study next in more detail.

### The relativity of the infection course and treatment effects

In the model, we assume that symptoms are pathogen-driven, thus treatment starts when the total bacterial density $B=B_{s}+B_{r}$ reaches a critical level Ω, the symptom threshold. Treatment administration lasts for *τ*_2_ days with a given antibiotic efficacy (kill rate) *A_m_* which represents the average net rate of antibiotic-induced bacterial killing at the infection site per unit of time. But what does Ω really mean? Should it be seen in absolute or relative terms?

**Fig. 3:**
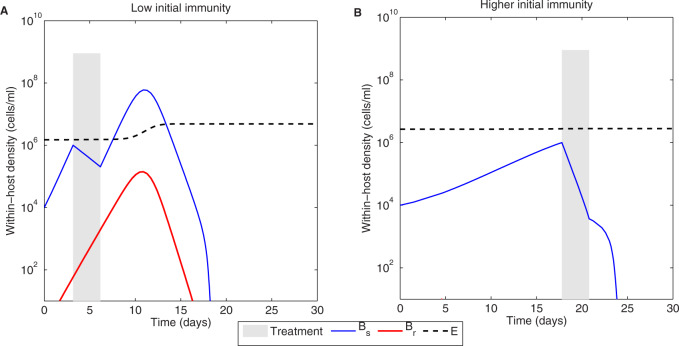
The same symptom threshold can result in different treatment outcomes depending on the initial immunity level. We simulated the model (see Eqs 1–3) assuming lower and higher initial levels of inducible immunity *E*_0_, which lead to different infection profiles: $B_{s}(t)$ and $B_{r}(t)$ bacteria depicted in blue and red lines, and immunity dynamics *E*(*t*) in black dashed line. (**A**) $E_{0}=E(0)=0.5E_{\mathrm{crit}}$. (**B**) $E_{0}=E(0)=0.89E_{\mathrm{crit}}$. The same treatment ($A_{m}=3,\tau_{2}=3$ days) is administered at the same density of bacteria within host $\Omega =10^{6}$, but with very different effect on infection. Here we used δ=10. All other parameters are the same as in Table 1.

First, when keeping all other parameters fixed, for a given infection, a higher Ω implies being at a later, more advanced stage of infection, where the pathogen level is surely higher, but the corresponding level of immunity is also expected to be higher. Second, when we shift patients, but keep the pathogen the same, the same Ω may mean the same pathogen level triggering treatment, but the corresponding level of immunity at such treatment onset will be different if the immune parameters are different between these two patients. Third, when we shift pathogens, in a slow-growing pathogen, starting from a given initial inoculum, the same Ω will be reached later than in a fast-growing pathogen. Thus, the same symptom threshold will mean a different point along their individual infection course. Fourth, when we shift pathogens and hosts, which is indeed the most common case, the critical point of treatment onset may be very hard to interpret. In fact, we may be dealing with the same instantaneous pathogen level that triggers symptoms, but with very different underlying infection history and kinetics (see Fig. 3). At the same pathogen level, a fast-growing infection, in contrast to a slower-growing one, likely harbors resistant bacteria, bound to be selected upon treatment and cause a relapse.

**Fig. 4:**
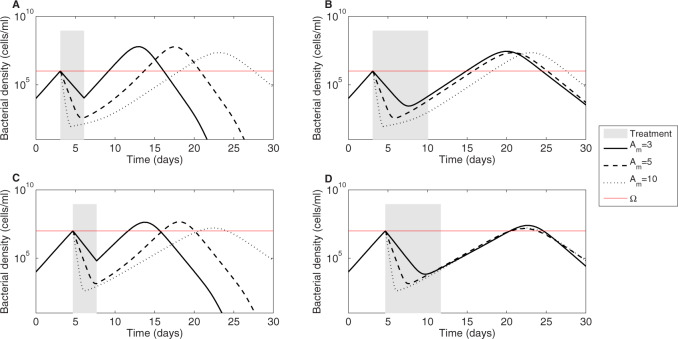
Treatment duration and timing effects on bacterial density dynamics over infection. We simulate the dynamics of the model (given in Eqs 1–3) and vary the timing of treatment (defined by Ω which influences *τ*_1_), the duration of treatment (*τ*_2_), and antibiotic kill rate (*A_m_*). (**A**) $\Omega =10^{5},\;\tau_{2}=3$ days. (**B**) $\Omega =10^{5},\;\tau_{2}=7$ days. (**C**) $\Omega =10^{6},\;\tau_{2}=3$ days. (**D**) $\Omega =10^{6},\;\tau_{2}=7$ days. Different lines denote $B(t)=B_{s}(t)+B_{r}(t)$ for different antibiotic kill rates *A_m_*, and the horizontal red line denotes the symptom threshold Ω. Other parameters as in Table 1.

Which treatment should be optimal then? How can one tailor antibiotic dose (kill rate) and treatment duration to such patient to patient variation? Indeed, this is rarely done. Typical treatment protocols involve fixed prescriptions, such as 3-day or 7-day treatments for a generic bacterial infection, with fixed antibiotic dosing (hence kill rate), independently of many patient characteristics or pathogen characteristics except patient’s age, sex and weight. Bacterial characteristics such as the number of bacteria, their growth rate and antimicrobial susceptibility to the prescribed drug are rarely measured (in part due to the difficulty of doing this in clinical practice). Such a lack of contextual information prior to treatment, may lead to different outcomes, even when the intention of treatment is to do no harm. We show that starting therapy at different bacterial densities and varying duration of treatment (3 vs. 7 days) results in the different global outcome (Fig. 4). Specifically, earlier treatment, when immune responses are just being developed, is likely to lead to higher relapses but a similar duration of infection, than the same treatment administered later. Increasing treatment duration prolongs the resolution of infection at both treatment timings, due to a higher selection of resistance. For short treatments, treatment results are more sensitive to the antibiotic kill rates. This happens because of the stronger role of the still-present sensitive bacteria in determining the competitive advantage of the resistant ones. For longer treatments, however, once the sensitive bacteria have been removed, the relative growth of the drug-resistant sub-population continues in a similar fashion unaffected by antibiotics. Thus, in longer treatments, resistant bacteria are typically causative of relapse, namely the relapse would not happen if the resistance were not there. Whereas in shorter treatments, relapses do not necessarily imply underlying drug-resistance, they may well indicate sensitive bacteria regrowth due to immune slowing down.

**Fig. 5:**
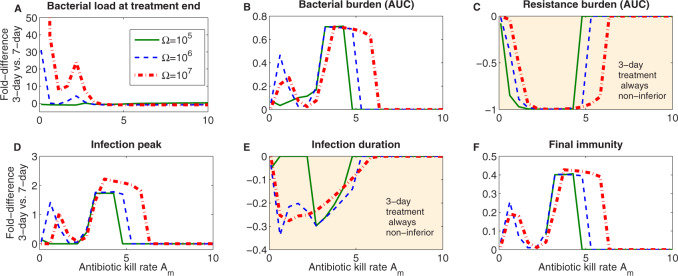
Quantitative comparison of short (3-day) versus longer (7-day) treatment along different metrics of success. For our mathematical model (Eqs 1–3) and varying Ω and *A_m_*, we calculate the relative fold-difference of target criterion of successful treatment $\Delta ={\text{Target}}_{3d}/{\text{Target}}_{7d}-1$. Positive and high values favour 7-day treatment, while negative and low values favour 3-day treatment, when the aim is to minimize a given target criterion.

Next, we compared 3- versus 7-day treatment quantitatively on different metrics of success (Fig. 5). The fold-difference in each target criterion between the two duration values, relative to the longer treatment (Δ), is plotted for a range of kill rates *A_m_* and for three timings along the same infection course. The comparison depends non-linearly and non-monotonically on the antibiotic kill rate. While the treatment timing does not qualitatively alter the comparison, it shifts the kill rates for a given relative superiority between long and short treatment. Close to the minimal inhibitory kill rates, and for suitably high kill rates, the two values of treatment duration give a similar performance (Δ≈0) in terms of determining the peak of infection, the bacterial burden and final immunity, suggesting that independently of Ω, the shorter treatment could be applied in most of these cases. Typically, for other kill rates, the 3-day treatment seems to be disfavored as it can yield up to about 20–40% higher bacterial burden and immune activation, and about 2-fold higher peak infection than 7-day treatment. There are only two exceptions where a short treatment is almost universally favored: resistance selection and infection duration (highlighted in Fig. 5C and E). This happens because resistance selection is a direct consequence of antibiotic treatment, and because infection duration is typically extended by any treatment, unless the kill rates used are extremely high. These graphs also display which infection characteristics covary the most, highlighting that targeting one of these could be sufficient to control the others.

**Fig. 6:**
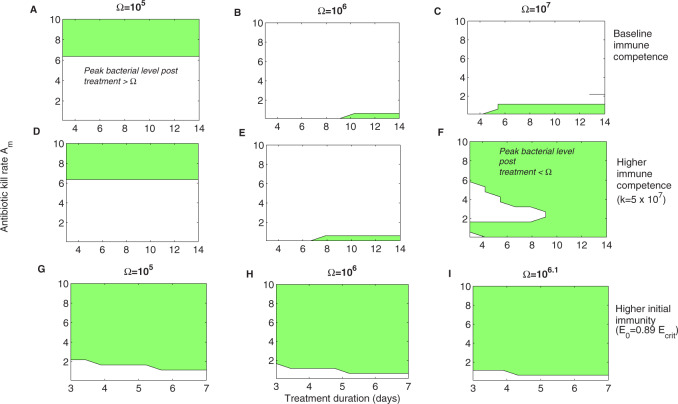
Treatment success versus treatment failure as a binary outcome. We plot the parameter regions where after treatment the peak bacterial density was lower than the one that triggered treatment in the first place, i.e., max⁡[B(t)]<Ω for all time $t>\tau_{1}+\tau_{2}$. We explored the effect of treatment timing along the same infection course. In (**A–C**), we used the default parameters. In (**D–F**), we simulated infection kinetics with lower *k*, half the original value in Table 1 ($k=0.5\times 10^{8}$), to illustrate the effect of higher immune competence in responding to infection. In (**G–I**), we used the same default parameters, but a higher initial level of immunity *E*_0_ ($E_{0}=0.89\times E_{\mathrm{crit}}$), which yields a much slower-growing infection but spread over longer time (see Fig. 3B)

While in the above comparison, detailed quantitative measures were used to compare treatment effectiveness, when prescribing antibiotics, doctors rarely have access to such quantitative picture of their patients, and randomized trials, typically rely on more crude but relevant measures such as recrudescence of symptoms. By using this binary measure in our simulations we next compared different antibiotic treatments on how they satisfy the simple criterion: max⁡[B(t)]<Ω during all time after treatment, i.e., for all $t>\tau_{1}+\tau_{2}.$ If satisfied, the condition indicates treatment effectiveness in resolution of symptoms, and, by logic, the patient should not require a second treatment. When this simpler measure is applied (Fig. 6), we find that for many combinations of antibiotic kill rate and duration of treatment, the shorter treatments are as effective as a longer treatment, if the kill rate applied is sufficiently high. The higher sensitivity of success (by this definition) to the kill rate than to treatment duration supports that whenever possible, shorter treatments could be used. However, we can always find regimes in which, by other criteria, shorter treatment is inferior to longer ones, further highlighting the challenge of finding a universally optimal treatment schedule (e.g. Fig. 5).

### Treatment timing and the immunity-resistance trade-off

The key to understanding how treatment duration affects infection dynamics is how bacterial load impacts the generation of the immune response. We assumed that immune response expansion is directly driven by the amount of bacteria and that immune response does not contract during the timescale of infection. This implies that the net growth rate of bacteria declines progressively due to the activation of the immune response, until it becomes negative for super-critical immunity. Thus, although parallel to a growing bacterial population, the probability of resistance emergence increases (Supplementary Figures S1-S2), the effective growth rate of any emerging resistant sub-population gets lower over time, limiting their eventual ascent and competitive advantage upon treatment. In this context, any treatment that does not clear bacteria but impairs immunity may lead to relapse. And the later such treatment occurs, the more likely this relapse is predominantly drug-resistant.

**Fig. 7:**
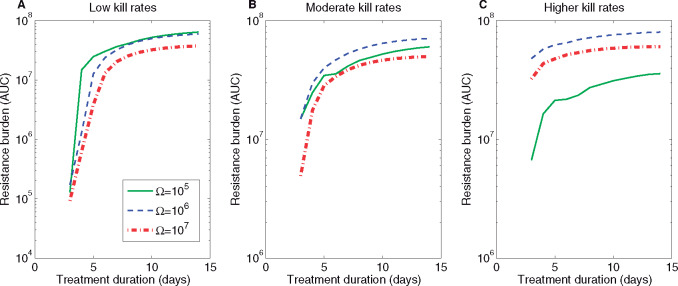
Antibiotic resistance selection as a function of treatment duration and pathogen density triggering symptoms Ω. We simulated model dynamics by varying antibiotic kill rate and the duration of treatment by averaging the relative resistance level for different kill rates. (**A**) $A_{m}\in [0.1,4]$, (**B**) $A_{m}\in [0.1,6]$ and (**C**) $A_{m}\in [0.1,10]$. We plot the mean resistance burden (AUC_*R*_) obtained in a 30 day period in model simulations with the default parameters (see Table 1) over 20 linearly spaced kill rates from the ranges specified. See Supplementary Fig. S6 for a similar analysis assuming a lower value of the mutation rate $m=10^{-6}$

The details of such immunity-resistance trade-off likely vary from infection to infection to determine which force is strongest. For our default parameter values, we find that increasing the length of treatment increases resistance selection irrespective of kill rates or treatment timing (Figure 7). However, the effect of increasing duration of treatment on ultimate resistance selection depends on the bacterial density where treatment begins. In particular, when treatment happens relatively early over the same infection course ($\Omega =10^{5}$ and $m=10^{-5}$), interference in the immune buildup and, thus, on the balance between *B_s_* and *B_r_*, can lead to a higher resistance burden than when treatment starts later ($\Omega =10^{6}$ and $m=10^{-5}$). This effect is pronounced at low kill rates (Fig. 7A), but gets reversed when higher kill rates are applied (Fig. 7C). At higher antibiotic efficacies, starting treatment at Ω=10^6^ yields a higher resistance burden than too early or too late treatments, indicating maximal selection potential upon intermediate timing, when immune activation is not yet complete, but with resistant bacteria already at sufficient levels. In general, the sensitivity of selection of resistance to treatment duration is higher towards the lower end of antibiotic kill rates (Fig. 7A) compared to the higher end (Fig. 7B-C), because once *B_r_* gain a definite advantage over *B_s_*, they subsequently grow unaffected by treatment. Next, since resistance selection depends on the actual mutation rate that generates resistance in the first place, we also examined the role of lower mutation rate on dynamics (Supplementary Fig. S6). When resistance generation becomes more difficult ($m=10^{-6}$), we observe that early timing restricts resistance more than later timing, indicating that when mutations are rare, treatments at lower pathogen densities are superior, in favor of the ‘hit fast’ principle (Supplementary Fig. S6). Yet, the pattern that increasing length of treatment increases selection of resistance, persists, also in this case, where late timings are the worst.

### Implications for optimal treatment

**Fig. 8:**
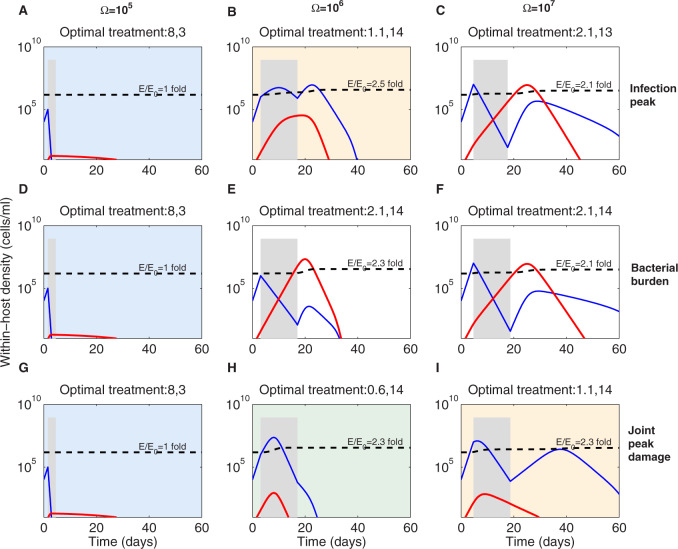
Optimal treatments and dynamics that minimize different target criteria. We varied antibiotic kill rates *A_m_* along 16 linearly spaced values between 0.1 and 8/day, and treatment duration *τ*_2_ between 3 and 14 days. For each combination (denoted as $A_{m},\tau_{2}$ at top of each panel) and each $\Omega =10^{6},10^{7},10^{8}$, we quantified the three metrics of success and finally searched for the optimal treatment that minimized the criterion (*B_s_* are shown in blue, *B_r_* in red, *E* in black dashed lines and the treatment interval depicted in gray shading). The ratio $E/E_{0}$ is computed at the end of the simulation period. In cases where more than one treatment emerged as optimal (Supplementary Fig. S5), we selected to show the one resulting from minimal total antibiotic-mediated killing. Sometimes the same treatment was favored, indicated by the background shading. The minimization criteria were: peak bacterial load (**A**–**C**), cumulative bacterial burden over 60 days (**D**–**F**), and the joint peak damage [product of peak $B_{s}(t)$, peak $B_{r}(t)$ and peak *E*(*t*)] (**G**–**I**). Other parameters are as in Table 1. For details, see also Supplementary Fig. S5

We find that shorter antibiotic courses would be better to constrain resistance risk in target bacterial populations, independently of treatment timing, with a higher relative benefit when treatment occurs at lower pathogen densities (Fig. 7). Antibiotic resistance however is only one indicator, among others, for defining successful treatment. We, therefore, used the model to probe treatment optimality by analyzing several alternative metrics of successful treatment as a function of antibiotic kill rate and treatment duration. We fixed the range of kill rates *A_m_* to 0.1−8 and of treatment duration to 3–14 days, in favor of limiting antibiotic exposure of a given patient as much as possible (due to potential side effects of the treatment). We compared the resulting optimal treatments across different treatment onset, as defined by pathogen load triggering symptoms Ω. Some criteria were unsurprisingly minimized in the extremes of maximally aggressive (long treatment with high kill rates) or no treatment—these included infection duration, immune activation and resistance selection (results not shown). For deeper analysis, we therefore selected (i) an *instantaneous* measure that captures the severity of infection (infection peak), (ii) a *cumulative* measure that integrates the area under the curve of infection balancing severity and duration (total bacterial burden) and (iii) a *joint* measure of three variables (the product between peak sensitive bacteria, peak resistant bacteria and peak immune response; therefore a balance between pathogen-induced pathology, resistance selection and immune-induced pathology) pathology (Fig. 8 and Supplementary Fig. S5).

We observe that optimal treatments tend to favor extremes in the two axes of control: either antibiotic kill rate or treatment duration, depending on the timing of administration. For early treatments, high-killing rates and short treatments are favored for all metrics of success (Fig. 8A, D and G). This suggests a *short and strong* principle for early treatment (Fig. 8A, D and G). In contrast, when treatment happens relatively later over infection, very long duration but low kill rates are favored, supporting a *mild and long* principle (Fig. 8B, C, E, F, H and I). Notice that the same treatment can have very different effects on the dynamics depending on timing. For example in Fig. 8B and I, the infection profile varies because treatment is applied at lower pathogen loads in one case and at higher pathogen loads in the other, with longer time to clearance in the latter, but more resistance selection in the former. It is interesting to observe that the optimal treatment that minimizes the joint peak damage, for intermediate timing (Fig. 8H), leads to a rapid clearance of infection, constrains substantially the ascent of resistance and reduces the activation of immunity to the minimum necessary and sufficient. However, this treatment, relying on mild kill rates and the ability of the immune response to grow during treatment, supports the notion that patients might need to accept getting slightly worse before getting better during treatment; a temporal trade-off hard to reconcile at first sight with the aim of clinical practice. Yet, with the exception of the early timing scenarios, in all other situations, this trade-off seems to apply, only the ‘getting worse’ is delayed to post-treatment relapses. This highlights that rationally confronting such trade-off is inevitable, although it may well-depend on patient-specific characteristics.

In summary, for all metrics, when treatment happens relatively early over the same infection course, the dominant leverage is on antibiotic kill rates, as they only are effective to prevent damage, by eliminating sensitive bacteria, restricting antibiotic resistance emergence and preventing any immune-activation that could lead to immunopathology. In contrast, when treatment happens relatively later and the damage is in process, strong kill rates can only make things worse, thus, milder killing but with longer treatments tends to be better, because this allows to control the damage, for example, by keeping the bacterial peak at most around the same level that triggered treatment in the first place. These treatments also keep antibiotic resistance in check because they counterbalance between high emergence probability but lower growth rate. At more advanced points along the infection course, the only option left is to harness the already-mounted defenses and limit any further increase in damage by treatment.

Overall, these results highlight fundamental motifs in antibiotic treatment success and failure that are likely to be shared across host–pathogen systems. Dissecting such motifs in terms of the interplay between treatment intensity, duration and host response in real infections remains key to improve treatment outcomes and reduce the risk of adverse effects, including the selection of antibiotic resistance.

## DISCUSSION

Antibiotics are essential to modern medicine and preserving their effectiveness is a global priority. Avoiding antibiotic overuse remains an important step in addressing the antibiotic resistance challenge [44, 45]. While some epidemiological studies have linked higher antibiotic use to higher levels of antibiotic resistance in host populations [46, 47], others found this link to depend on the drug with no significant correlation for many drugs [48]. It is clear, though, that even at the single host level, we need a better understanding of how individual infection processes and treatment parameters affect resistance dynamics. In this study, we examined the optimality of antibiotic treatment, taking into account several infection target criteria, and expanding on the previously neglected aspect of treatment start time over the infection course (but see [27, 49, 50]).

We found several parameter regimes and target criteria by which short (3 days) treatment is non-inferior and even superior to longer (7 days) treatment, lending support to reduced courses of antibiotics to achieve similar clinical outcomes [43]. Our results suggest that among treatments far away from the aggressive spectrum, those that maintain slightly higher pathogen densities within the host can minimize the selection of resistance, similar to other studies [42, 51]. In our parameter regime, this effect comes mainly from heightened indirect competition between drug-sensitive and drug-resistant populations due to higher immune activation expected at higher bacterial loads. In the model’s general mechanistic structure and in reality, this effect is also compounded by resource-mediated competition (e.g. the role of the within-host carrying capacity).

We also observe that optimal kill rate-duration combinations vary, depending on the time point of treatment, infection criterion to optimize and host characteristics. This difficulty in drawing general principles for a multi-factorial problem is not new. At the epidemiological level, it is also being recognized that ranking antibiotic treatment protocols is highly dependent on methodological factors, e.g., the criterion of choice for comparison [52]. It is likely that a better understanding of how individual infection processes and treatment parameters affect resistance dynamics and health across multiple biological scales will help sharpen expectations and adapt treatment to meet the selected goals.

Although we find a general qualitative pattern of optimal treatments, supported by the model, shifting from ‘short and strong’ for early treatments to ‘mild and long’ for later treatments implementation of such principle may be difficult in practice. Distinguishing patients that are less tolerant (early treatment seekers) from those that are more tolerant (later treatment seekers) may be hard, and thus, adjusting antibiotic kill rates and treatment duration to individual patients may be an impossible task. However, our results suggest that strongly bactericidal versus more bacteriostatic antibiotics could be used in different patient groups if they can somehow be categorized based on the point along their infection course at which they report symptoms. This remains a conjecture since we did not actually model the impact of bacteriostatic drugs on infection dynamics. 

In our model, resistant bacteria, emerging at low frequency in an infection, are more likely to go extinct in high-host immunity settings (Fig. 3), where drug-sensitive competitors and high levels of immune control reduce the absolute fitness of the resistant mutants. This within-host pattern can have implications for dynamics of resistance at the higher scale of populations, and has been argued as a plausible hypothesis for drug resistance in malaria parasites in high transmission versus low transmission settings [53]. Within-host competition remains to be studied deeper analytically, in all its dimensions, beyond the ones covered here, and beyond simulations, as it is a key factor shaping the evolution of drug resistance in infectious diseases. Nested models connecting the within- to the between-host levels are crucial for this aim [23, 52], but in the field of antibiotic resistance, future models need to properly account also for variation in timing and duration of treatment in individual hosts.

There are a number of potential extensions to our modeling analysis. We did not study the role of transmitted pre-existing resistance; starting the infection with already pre-existing resistant variants can be understood in this model via an increase in mutation rate, which would generate resistance even faster within the host. This is likely to disfavor longer treatments [15]. We ignored pharmacokinetics and pharmacodynamics of the antibiotics which may also on their own add uncertainty in defining optimal treatment duration. However, focusing on the net kill rate at the infection site, the interplay between the three major axes of treatment becomes more evident and easier to understand. Opting for a more parsimonious representation of antibiotic efficacy (only one parameter for kill rate instead of four required to specify a full pharmaco-dynamic function [54]), we allow for the same kill rate to be attained with different antibiotics, each possibly with a different concentration, hence adding generality to our formulation. Other studies [22, 23, 25, 29], motivated by *in vitro* data, have included explicit resource dynamics and explicit antibiotic concentrations via Hill functions in their models. It is not clear how these functions apply to a complex *in vivo* situation, where ultimately how microbial death rates compare to growth rates matters. Thus, since much remains unknown about *in vivo* dynamics of bacteria, resources and drugs during resistance generation, we decided to represent essential processes in the simplest way (exponential growth and decline), so that experiments or future *in vivo* data can most directly inform the model parameters, a practice widely adopted in viral kinetics models [55, 56].

We opted for a hybrid approach to simulate the generation of drug-resistant variants. Given that we consider a relatively high mutation rate, drug-resistant mutants are expected to be present often at the start of treatment (but see Supplementary Fig. S2). Therefore, most of our results are likely to remain valid even if the generation of mutants is described deterministically (results not shown). We considered a single mutational step to complete resistance. This is likely a simplification but serves as a benchmark for a worst-case scenario in empiric therapy, where complete resistance means that this treatment is ineffective in the short term for the patient, and urgent antimicrobial susceptibility characterization of the underlying pathogen is needed. In particular, when the antibiotic kill rate is very high, our model provides a large advantage to drug-resistant bacteria, which may be lower in most real-life infections, and generate lower relapses than the ones predicted here. In our case, antibiotic treatment alone cannot clear a resistant-only infection—a phenomenon observed more in critical clinical cases [57], thereby emphasizing competition with drug-sensitives and immune-mediated killing as crucial co-factors for infection control.

Furthermore, resistance is likely to be acquired in gradual mutational steps [4, 58] where transient phenotypes in fitness cost and antibiotic susceptibility may interact differently with treatment, reducing *B_r’_*s competitive advantage, or altering their interplay with the immune system. Similarly, spatial heterogeneity of an infection was not studied, focusing on infection that develops more or less homogeneously at a single primary site. Spread of bacteria in different tissues, and unequal distribution of antibiotics in such within-host compartments, may select for resistance at different rates, effectively resulting in different reservoirs of infection, each with their own local dynamics, contributing to more complex symptoms and infection profiles.

In our model, host immunity is a key player in treatment success, resonating with previous studies [22, 27, 29]. How the dynamics of immune response, both innate and adaptive, depends on the presence of infection in humans is not understood and remains an active area of investigation. Variation according to type of infection is expected [59] and different aspects of host susceptibility must be accounted for in an integrative framework [60]. The kinetics of most acute bacterial infections in humans have not been accurately measured (but see [34, 61]). One critical parameter in our analysis was the time when treatment starts (which in the model was strictly determined by the bacterial density). Physiological factors driving patient symptoms, tolerance to infection [62], pathology and treatment onset remain unclear, and are likely to widely vary between individuals, as evidenced, for example, by differences in microbiologic confirmation at baseline across patients with the similar symptoms [63]. All these aspects require attention in the future.

Deeper understanding of immune processes will not only aid the antimicrobial resistance challenge but also help improve the management of infection, immune suppression, cancer and other diseases, in a more personalized way. Recently, individual disease trajectories have been introduced as a powerful way to represent the dynamic nature of infection and its effect on health in single hosts [64], where multiple infection and health variables are plotted and analyzed together as they covary during disease progression. Such approaches could be applied also to inform rational antibiotic therapies, by revealing the critical infection stages or host types where the benefits of treatment would be maximal. Empirical quantification of mechanisms regulating bacterial loads and bacteria-specific immune responses in tissues in human infections will definitely test and constrain mathematical models. Only this will enable robust predictions about interventions.

## Supplementary data

Supplementary data is available at *EMPH* online.

## Supplementary Material

eoaa033_Supplementary_Data
